# Subconjunctival injection of human umbilical cord mesenchymal stem cells alleviates experimental allergic conjunctivitis via regulating T cell response

**DOI:** 10.1186/s13287-023-03484-4

**Published:** 2023-10-02

**Authors:** Dongli Li, Qingjian Ou, Qi Shen, Michael Mingze Lu, Jing-Ying Xu, Caixia Jin, Furong Gao, Juan Wang, Jingfa Zhang, Jieping Zhang, Jiao Li, Lixia Lu, Guo-Tong Xu, Haibin Tian

**Affiliations:** 1 Department of Ophthalmology of Tongji Hospital, Laboratory of Clinical and Visual Sciences of Tongji Eye Institute, School of Medicine, Tongji University, 389 Xincun Road, Shanghai, 200065 China; 2 Department of Ophthalmology, Shanghai General Hospital (Shanghai First People’s Hospital), Shanghai Jiao Tong University, Shanghai, 200080 China; 3 Department of Physiology and Pharmacology, TUSM, Shanghai, 200092 China; 4 The Collaborative Innovation Center for Brain Science, Tongji University, Shanghai, 200092 China

**Keywords:** Allergic conjunctivitis, Human umbilical mesenchymal stem cells, Immunomodulatory, Subconjunctival injection, Th2 cells

## Abstract

**Background:**

T helper 2 (Th2) cells are thought to play critical roles in allergic conjunctivitis (AC). They release inflammatory cytokines to promote an allergic response in AC. Due to individual heterogeneity and long-term chronic management, current therapies do not always effectively control AC. Mesenchymal stem cells (MSCs) have been shown to be effective in treating allergy-related disorders, but it is unclear how exactly the Th2-mediated allergic response is attenuated. This study aims to elucidate the therapeutic effect and mechanism of the human umbilical cord MSCs (hUCMSCs) in a mouse model of experimental AC (EAC).

**Methods:**

A mouse EAC model was established by inoculating short ragweed (SRW) pollen. After the SRW pollen challenge, the mice received a single subconjunctival or tail vein injection of 2 × 10^6^ hUCMSCs, or subconjunctival injection of hUCMSCs conditioned medium (hUCMSC-CM), and dexamethasone eye drops was used as positive control; subsequent scratching behavior and clinical symptoms were assessed. Immunostaining and flow cytometry were carried out to show allergic reactions and the activation of CD4 + T cell subsets in the conjunctiva and cervical lymph nodes (CLNs). Gene expression was determined by RNA-seq and further verified by qRT-PCR and Western blot. Co-culture assays were performed to explore the regulatory role of hUCMSCs in the differentiation of CD4 + naive T cells (Th0) into Th2 cells.

**Results:**

Subconjunctival administration of hUCMSCs resulted in fewer instances of scratching and lower inflammation scores in EAC mice compared to the tail vein delivery, hUCMSC-CM and control groups. Subconjunctival administration of hUCMSCs reduced the number of activated mast cells and infiltrated eosinophils in the conjunctiva, as well as decreased the number of Th2 cells in CLNs. After pretreatment with EAC mouse serum in vitro to mimic the in vivo milieu, hUCMSCs were able to inhibit the differentiation of Th0 into Th2 cells. Further evidence demonstrated that repression of Th2 cell differentiation by hUCMSCs is mediated by CRISPLD2 through downregulation of STAT6 phosphorylation. Additionally, hUMCSCs were able to promote the differentiation of Th0 cells into regulatory T cells in CLNs of EAC mice.

**Conclusions:**

Subconjunctival injection of hUCMSCs suppressed the Th2-allergic response and alleviated clinical symptoms. This study provides not only a potential therapeutic target for the treatment of AC but also other T cell-mediated diseases.

**Supplementary Information:**

The online version contains supplementary material available at 10.1186/s13287-023-03484-4.

## Introduction

Allergic conjunctivitis (AC) has been characterized as a bilateral ocular surface inflammatory response resulting from exposure to allergens such as pollen, house dust mites (HDM), animal dander, or some molds [1]. There are four types of allergic conjunctivitis: vernal and atopic keratoconjunctivitis (chronic), and perennial and seasonal allergic conjunctivitis (acute). The latter two are the most common types, leaving 30–40% of the general population with diminished quality of life [2, 3]. Refractory and chronic cases may lead to vision-threatening ocular surface complications. They may also deprive corneal allografts of immune privileges and increase immune rejection [4–6]. The main symptoms of AC are itching, redness, and increased tear production.

Of the several immune cells implicated in the occurrence or progression of allergic diseases (e.g., mast cells, T cells, B cells, dendritic cells), the inappropriate activation of T helper 2 (Th2) cells has been recognized as a critical mechanism in various allergic reactions [7]. After being exposed to a conjunctival allergen, dendritic cells (DCs) migrate into the cervical lymph nodes (CLNs) to initiate the proliferation of naive T cells (Th0) and differentiation into Th2 cells. Cytokines produced by Th2 cells, namely interleukin (IL)-4, IL-5, and IL-13, favor the activation and stimulation of B cells to produce IgE. Mast cells (MCs) and basophils have high-affinity IgE receptors which, when bound with IgE, “sensitizes” the cells [8]. Upon re-exposure to the allergen, bound IgEs on a sensitized MC undergo crosslinking and leads to the degranulation of the cell. The subsequent release of histamine, cytokines, lipid mediators, and chemokines, contribute to the clinical symptoms of AC [9]. In addition, there is growing evidence of other pathways contributing to Th2-dominant allergic inflammation, such as Th17 cell-derived IL-17 [10, 11], as well as innate immunity. It was recently reported that, during AC pathogenesis, elevated group 2 innate lymphoid cells produce cytokines such as thymic stromal lymphopoietin, IL-25, and IL-33 that enhance Th2 response and induce inflammation [12]. On the other hand, regulatory T cells (Treg) cells have been acknowledged as a suppressor of Th2-dependent allergic inflammation and mast cell degranulation, facilitated by the production of IL-10 and transforming growth factor-β (TGF-β). Dysregulation of Treg cells could lead to allergic sensitization and allergic disease [13].

Generally, pharmacological treatments for acute and chronic forms of AC include a variety of topical and oral agents (e.g., short-acting beta-agonists, antihistamines, leukotriene receptor antagonists, and corticosteroids). However, these compounds only provide temporary symptomatic relief, since they cannot directly address the root cause. Furthermore, they are limited by systemic side effects and drug resistance [14]. Other potential treatments, such as allergen-specific immunotherapy, JAK/STAT inhibitors, and Bruton’s tyrosine kinase (BTK) inhibitors, are being evaluated in clinical trials [15, 16]. Mesenchymal stem cells (MSCs) have emerged as a promising alternative for various allergic and autoimmune diseases [17]. Su et al. found that the conditioned medium (CM) from bone marrow-derived MSCs stimulated by tumor necrosis factor-a (TNF-α) alleviates experimental AC (EAC) by suppressing the activation of MCs, reducing the number of inflammatory cells, and the levels of related factors (e.g., TNF-α, IL-4, NF-κB) [18]. Using a mouse model of ovalbumin-induced airway inflammation, Kavanagh and Mahon et al. reported that systemic administration of allogeneic MSCs inhibited IL-13 and IL-4 production, and activated Treg cells [19]. Shin et al. [20] demonstrated that human umbilical cord-derived MSCs (hUCMSCs) attenuated inflammation in mouse asthma models and downregulated IL-5 and IL-13 production in Th2 cells from mice. The available evidence indicates that MSCs have the capacity to suppress Th2 function in Th2-dominated inflammation [21].

However, the mechanisms underlying the regulation of Th2 cells by MSCs in AC are still poorly understood. In addition, there is limited literature demonstrating the role and secretion profile of MSCs in AC. Herein, we investigated the therapeutic functions of hUCMSCs in an SRW-induced mouse EAC model and explored the mechanisms behind the suppression of T lymphocyte subset differentiation by hUCMSCs. In addition, the different delivery routes of hUCMSCs were also evaluated. We demonstrated that subconjunctival administration of hUCMSCs was able to alleviate symptoms of EAC, and the mechanism involved hUCMSCs secreting CRISPLD2(Cystine-rich secretory protein and two LCCL domains) to inhibit the differentiation of naive T cells into Th2 cells; our results provide new insights into the immunoregulatory mechanism mediated by MSCs in AC and other T cell-mediated allergic diseases.

## Materials and methods

### Animal preparation

The BALB/c strain is preferred for the SRW-EAC model because the mouse has a Th2-prone nature and female mice tend to have a more robust inflammatory response at the clinical level, as compared to males [22]. Thus, six-week-old female BALB/c mice (Laboratory Animal Center of Tongji University) were used in this study. All experiments on animals were approved by the animal experimentation ethics committee of Tongji University (Approval NO. TJAA09620101). Principles of animal research conformed to the guidelines of ARVO Statement for the Use of Animals in Ophthalmic and Vision Research. All mice were maintained on a regular diurnal lighting cycle (12 h light and 12 h dark) with access to food and water. Mice were randomly allocated to experimental groups and no blinding method was used for cell transplantation. There was no animal exclusion criterion.

### hUCMSCs culture, identification, and stimulation

The hUCMSCs were obtained from the Eastern Union Stem Cell and Gene Engineering Co., Ltd. (China). Informed consent was obtained by Eastern Union Stem Cell and Gene Engineering Co., Ltd. Cells were cultured in Dulbecco’s modified Eagle’s medium (DMEM)/F12 medium (11320033, ThermoFisher Scientific, USA) containing 10% FBS (10099141C, ThermoFisher Scientific, USA) at 37 °C, 5% CO_2_.

The cells were identified according to our previous study [23]. Briefly, hUCMSCs were induced to differentiate into adipocytes, osteoblasts, and chondrocytes. For adipogenesis, the cells were cultured in adipogenic induction medium (DMEM/F12 supplemented with 10% FBS, 10^−7^ M dexamethasone (D4902, Sigma, St. Louis, MO), 100 μΜ indomethacin (I7378, Sigma), 100 μM 3-isobutyl-1-methyl-xanthine (I7018, Sigma) and 10 mg/L insulin (T8221, TargetMol, USA)), and the medium was refreshed every 2 days. Two weeks later, the cells were fixed with 4% paraformaldehyde (PFA, E672002, Sangon Biotech, China) and stained with Oil-Red O (O1391, Sigma). For osteogenesis, the cells were maintained in osteogenic induction medium (DMEM supplemented with 10% FBS, 10 mM β-glycerol phosphate (G9422, Sigma), 50 μM L-ascorbate-2-phosphate (A4544, Sigma) and 5 ng/mL recombinant human bone morphogenic protein-2 (HZ-1128, HumanZyme, Chicago, IL)), and the medium was changed with fresh one every 2 days. One week later, the cells were examined for alkaline phosphatase (AKP) activity by vector blue alkaline phosphatase substrate kit III (SK-5300, Vector, Burlingame, CA). For chondrogenesis, the cells were cultured in chondrogenic induction medium includes DMEM supplemented with 10% FBS, 10 ng/mL TGF-β1 (100–21, PeproTech), 0.1 mol/L dexamethasone (Sigma), 50 mg/L L-ascorbate-2-phosphate (Sigma) and 50 g/L ITS (41400045, ThermoFisher Scientific, USA). The cells were cultured for 2 weeks and fixed in 4% PFA, and stained with toluidine blue sodium (T3260, Sigma).

For cell membrane marker identification, hUCMSCs were detached and dissociated into single cells with 0.25% trypsin / 0.53 mM EDTA buffer (E607002, Sangon Biotech, China), then resuspended in PBS containing antibodies against CD105, CD90, CD73, CD44, CD29, CD34, CD45, and MHCII (BD Bioscience, San Diego, CA). Flow cytometry were analyzed by FACS Aria II instrument using Cellquest software (BD Bioscience).

For clarifying the immunoregulatory effect of hUCMSCs, the cells were pretreated with serum obtained from normal or EAC mice with 10% concentration in DMEM/F12 medium supplemented with penicillin and streptomycin antibiotic solution (15140148, ThermoFisher Scientific, USA) for 24 h at 37 °C in a 5% CO_2_ humidified atmosphere. The hUCMSCs were cultured to 90% confluence, the cultured medium was refreshed, and 24 h later, the hUCMSC-CM was collected.

### Establishment of mouse EAC model and administration of hUCMSCs

Animals were randomized using a computer based random order generator and divided into four groups (six animals/group); for the establishment of the EAC model, BALB/c mice were immunized with 50 µg of short ragweed (SRW) pollen (Greer Lab, Lenoir, NC USA) by footpad injection at day 0 and a secondary injection at day 5. Then, the mice were injected intraperitoneally with pentobarbital sodium (1% v/v) for anesthesia. Next, by using eye drops, 1.5 mg of SRW pollen dissolved in 10 µL of PBS was administered to each eye once a day from day 10 to 15. For the experimental group, at day-10 following the SRW pollen challenge, 2 × 10^6^ cells were delivered in 30 μL of PBS via subconjunctival injection, or in 200 μL of PBS via intravenous injection. For DEX group, the mice were treated with 10 μL dexamethasone (EAC, TobraDex Dexamethasone eye drops from Alcon), four times per day. For hUCMSC-CM group, 30 μL hUCMSC-CM was delivered via subconjunctival injection.

For each animal, three investigators were involved for different roles, respectively, and animals that failed the injection were excluded in the analysis, including the SRW pollen were not completely injected into the animals, the cells were not accurately injected into tail vein, and/or the subconjunctivally injected cells were leaked from injected site. The signs of immediate hypersensitivity responses and instances of scratching in the 20 min after the topical-SRW pollen challenge were evaluated. Total clinical signs were scored based on chemosis, conjunctival redness, lid edema, and tearing, and each was graded from a score of 0 to 3 (0 = absent; 1 = mild; 2 = moderate and 3 = severe), the sum of the scores for each item is used to evaluate EAC symptom and calculate the *P* value [24]. Twenty-four hours after the last challenge, the mice were euthanized by cervical dislocation after CO_2_ anesthesia, and the blood of EAC mice was collected and centrifugated, and the supernatant was labeled as EAC mouse serum and stored at − 80 °C for pretreatment of hUCMSCs in vitro.

To evaluate whether the intravenously injected hUCMSCs migrated to the conjunctiva, shControl-hUCMSCs expressing exogenous ZsGreen were intravenously injected into the mouse EAC model. Twenty-four hours later, conjunctivas were collected and cryosections were prepared. Samples were observed under fluorescence microscope (Olympus IX73; Olympus, Tokyo, Japan). Samples with subconjunctival injection of shControl-hUCMSCs were used as the positive control.

### Histologic analysis of conjunctiva and CLNs

The eyes with eyelids and conjunctival tissue attached, and the CLNs were collected on day-15 following the SRW pollen challenge. For dissection of conjunctiva, the mouse eyes with eyelids were isolated and the hair around the eyelids was trimmed using micro-scissors and forceps under a dissecting microscope; tissues were fixed with 4% paraformaldehyde and embedded in optimal cutting temperature compound (VWR, Suwanee, GA). Cryosections (10 μm thickness) of eyeballs and CLNs were prepared with a cryostat (Leica CM 1950, Germany); the infiltrated eosinophils were detected by Giemsa staining (C0131, Beyotime, China), and MCs were stained by toluidine blue staining (G3670, Solarbio, China). The sections were visualized with a microscope system (Olympus IX73; Olympus). For isolation of lymphocytes, spleen and CLNs were collected from each mouse. Spleen and CLNs were cut into small pieces and then were triturated with the flat end of the syringe plunger and filtered through 70-μm cell strainer. Red blood cells (RBC) were lysed by RBC lysis buffer. After centrifugation, the cells were resuspended in 500 μL fluorescence-activated cell sorting (FACS) buffer. The fluorochrome-conjugated primary antibody cocktail or fluorochrome-conjugated isotype-matched control antibodies was added into the FACS buffer for cell staining and flow cytometric analysis.

For immunostaining, sections were incubated with primary antibodies against CD4, IL-4, IL-5, IL-10, IL-17, IL-13, FOXP3, IFN-γ, and Cytokeratin7, then incubated with Alexa Fluor 488 or 555–conjugated anti-rabbit and mouse IgG antibody (Jackson ImmunoResearch Labs). Antibodies are listed in Additional file 1: Table S1. Afterward, nuclei were stained with DAPI (Vector Laboratories, Burlingame, CA). The samples were then examined by fluorescence microscope (Olympus IX73).

### RNA isolation and quantitative real-time PCR

Total RNA was extracted and reverse transcription was performed using Primescript™ RT Master Mix kit (Takara, Shiga, Japan). qRT-PCR was performed in a Chromo4 instrument cycler (Bio-Rad, Hercules, USA) using Superreal Premix plus kit (Tiangen Biotech, Beijing, China). PCR amplification was carried out with the following cycling parameters: denaturation at 95 °C for 5 min, followed by 40 cycles of 95 °C for 10 s, 60 °C for 30 s. Primer sequences (Synthesized by Sangon Biotech, China) were listed in Additional file 1: Table S2.

### Flow cytometry

Fresh CLNs were dissociated, resuspended in FACS buffer, and filtered through a 70-μm mesh cell strainer (Corning, NY, USA). The single-cell suspensions were labeled with anti-CD4-FITC (11-0041-81, eBioscience) or anti-CD25-PE-Cyanine7(25-0251-81, eBioscience) antibodies. Cells were washed with pre-chilled PBS to remove unbound antibodies. Fixation and permeabilization were performed with the fixation and permeabilization kit (88-8824-00, eBioscience). After washing once in PBS and twice in buffer, cells were incubated with anti-IFN-γ-PE (12-7311-82, eBioscience) or anti-IL-4-APC (17-7041-81, eBioscience) antibodies. FOXP3 was labeled with Brilliant Violet 421™ anti-mouse FOXP3 Antibody in True-Nuclear™ Transcription Factor Buffer (126419, Biolegend). Flow cytometric analysis was performed on a flow cytometer (Beckman Coulter, CA, USA), and data were analyzed with Flow Jo 10.7.1 software or CytoExpert software (Beckman Coulter).

### RNA sequencing

Following the Illumina mRNA-seq protocol, pooled RNA libraries of the cells were established, with 50 ng of RNA from hUCMSCs treated with serum from EAC mice or normal mice. Sequencing was performed by the MAJORBIO company (Shanghai, China). Filtering and quality control of the raw reads from RNA-seq was carried-out using FastQC. The clean reads were mapped to reference sequences using SOAP2 aligner. Gene expression levels were calculated using the TPM method. Log_2_ fold change (FC) of TPM (hUCMSCs treated with EAC serum) / TPM (hUCMSCs treated with normal serum) was used to identify differentially expressed genes (DEGs) between these two groups. Only those genes indicating log2|FC|> 0.585 and adjusted *P* < 0.05 were regarded as DEGs.

### Assembly of lentivirus to knockdown crispld2

Lentiviral pLVX-shRNA2-ZsGreen1 (Takara) vector was used to prepare lentivirus. The packaging plasmids psPAX2 and pMD2.G were used. The shRNA target sequences were as follows: *Crispld2*-RNAi-1: CGTCAGATGTGACACCAAGAT; *Crispld2*-RNAi-2: GCAGCTGCAGGAACAACTTGT; *Crispld2*-RNAi-3: GGAGTACATGACCTGGGATGA. Non-targeting sequence construct was used as the control: GCGCGATAGCGCTAATAATTT. HEK293T cells were transfected with vectors. Individual supernatants containing the assembled virus were harvested at 48 h and used to infect hUCMSCs. The positively transfected cells were sorted by FACS based on ZsGreen expression. Reduced expression of target gene *Crispld2* at the transcript level was determined by qRT-PCR. The most effective shRNA sequence out of the three was selected.

### T cell proliferation assay

CD4 + T cells from spleen cells of BALB/c mice were isolated with a negative-selection magnetic cell separation (MACS) approach. MACS separation was performed using the Dynabeads™ Untouched™ CD4 + T Cell Isolation Kit (11415D, ThermoFisher Scientific) and LS column (130-042-401, Miltenyi Biotec). 1 × 10^5^ hUCMSCs were seeded in a 6-well culture plate overnight. Isolated T cells were labeled with Carboxy-fluorescein succinimidyl ester (CFSE) and co-cultured with hUCMSCs by direct contact pattern in the presence of CD3/CD28 activation beads. The proliferation index of T cells was assessed based on CFSE dye dilution rate after 3 days of co-culturing.

### Differentiation of Th0 cells

Th0 cells (CD4 + CD25-CD62LhighCD44low) from lymph nodes and spleen of BALB/c mice were isolated and purified by flow cytometry. Then, in 12-well plates with humidified 5% CO_2_ at 37 °C, Th0 cells (2 × 10^6^/ml) were treated with Dynabeads™ Mouse T-Activator CD3/CD28 (11456D, ThermoFisher Scientific) in the presence of IL-2 (10 ng/ml). hUCMSCs were co-cultured with T cells at a ratio of 1:5 by direct contact pattern. For Th2 differentiation, anti-IFN-γ antibody (10 μg/ml) and IL-4 (20 ng/ml) were added at the start of co-culture. For Treg differentiation, anti-IFN-γ antibody (10 μg/ml) and TGF-β1(20 ng/ml) were added at the start of co-culture. To assess the polarization of Th2, cells were stimulated with a cell activation cocktail containing PMA, ionomycin, and Brefeldin A (423303, Biolegend) for 6 h. The cells were then fixed and permeabilized with Cyto-Fast™ Fix/Perm Buffer Set (426803, Biolegend) and stained with anti-CD4-FITC, anti-IFN-γ-PE, or anti-IL-4-APC antibodies. Treg cells were fixed and permeabilized with True-Nuclear Transcription Factor Buffer Set and stained with anti-CD4-FITC, anti-CD25-PE-Cyanine7, and anti-FOXP3-Brilliant Violet 421™ antibodies (126419, Biolegend). After staining, cells were evaluated by flow cytometry.

### Western blot analysis

The CD4 + T cells co-cultured with hUCMSCs were lysed in RIPA lysis buffer (P0013B, Beyotime, China) containing protease and phosphatase inhibitors (C0001 and C0004, TargetMol, USA). A total of 10 µg protein was separated by 10% SDS-PAGE and transferred onto a polyvinylidene difluoride (PVDF) membrane (Millipore, Billerica, MA, USA). After blocking with 3% BSA in PBS for 2 h, the membrane was incubated with primary antibodies against phospho-signal transducer and activator of transcription (STAT) 6 (1:1000, Abcam, ab263947), STAT6 (1:1000, Proteintech, 10253-2-AP), or GAPDH (1:5000, Proteintech, 60004-1-Ig) for 12 h, then incubated with anti-rabbit secondary antibody conjugated to horseradish peroxidase (1:5000, Proteintech, SA00001-2) for 1 h at room temperature. The blots were visualized with a chemiluminescence imaging system (Tanon 5200, Shanghai, China) and quantified using ImageJ (Version 1.48v).

### Statistical analysis

GraphPad Prism (Version 9.3.0) was used for statistical analysis. All quantitative data were recorded as mean ± SD. Statistical differences between two datasets were evaluated using Student’s t test. Multi-group comparisons were analyzed by one-way ANOVA and post hoc Bonferroni’s test. *P* values less than 0.05 was considered significant. All experiments were repeated in triplicates.

## Results

### Subconjunctival injection of hUCMSCs alleviates EAC

**Fig. 1:**
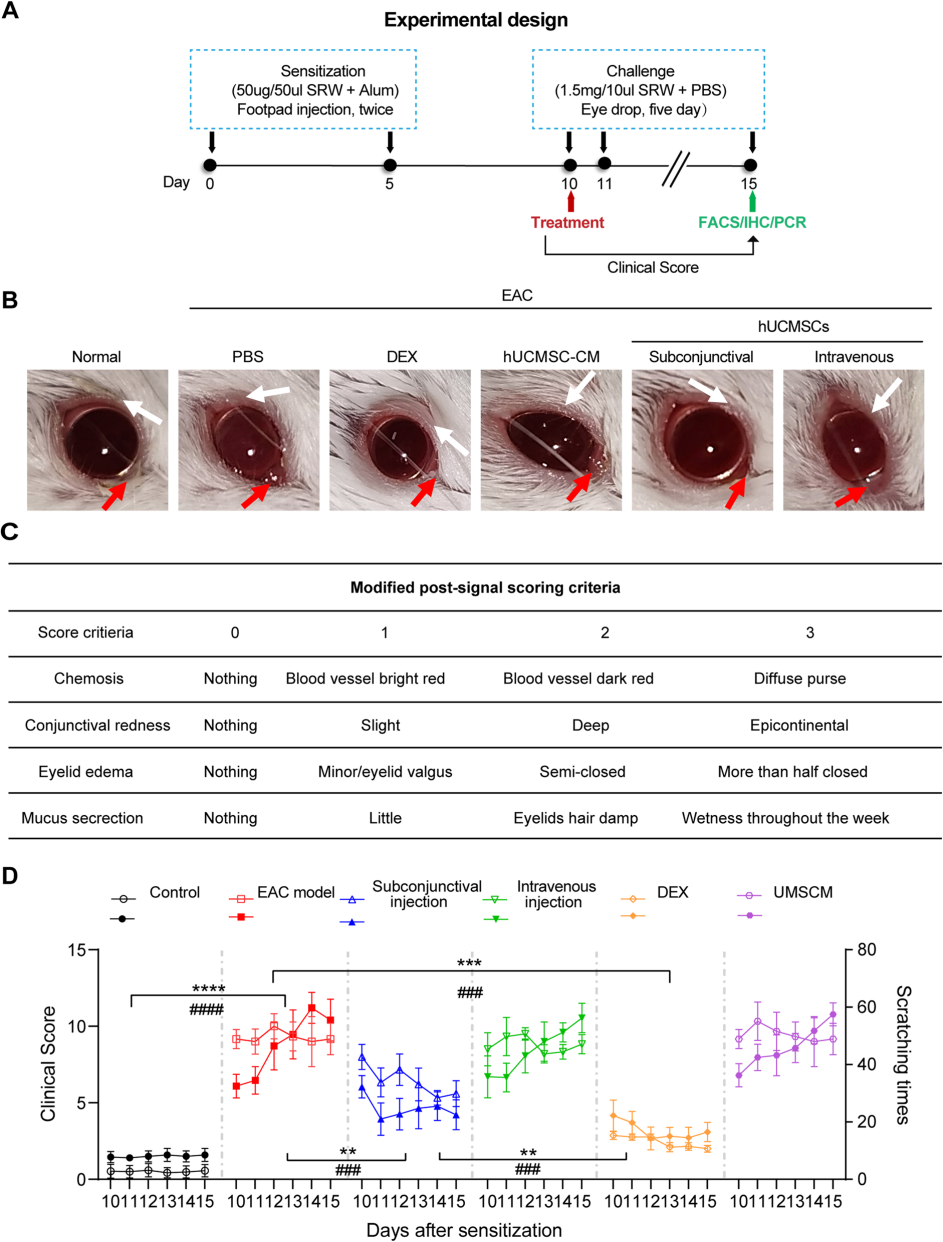
Subconjunctival injection of hUCMSCs alleviates EAC. **A** Schematic depiction of the experimental design. **B** Representative images of ocular symptoms in differential groups after the last SRW pollen challenge; white arrow pointed to the eyelid edema, and the red arrow pointed to the mucoid discharge and chemosis. **C** A modified scoring system (total score: 0–12), including conjunctival redness (0–3), chemosis (0–3), lid edema (0–3), and tearing (0–3), was used to assess clinical signs of AC. **D** The instances of scratching responses in different groups (hollow symbols) were evaluated at the indicated time points after being challenged with SRW pollen solution (*n* = 6). Mean clinical scores (solid symbols) showed the severity of conjunctivitis in different groups at different time points (*n* = 6). Results were expressed as the mean ± SD, **P < 0.01, ***P < 0.001 in clinical scores; ###*P* < 0.001 in scratching responses

hUCMSCs demonstrated fibroblast-like morphology and possessed the capacity to differentiate into adipocytes, osteocytes, and chondrocytes (Additional file 2: Fig. S1A), as well as expressed the cell surface markers CD105, CD90, CD73, CD44, and CD29, but not CD34, CD45, and MHCII (Additional file 2: Fig. S1B).To investigate whether different routes of administration affected the therapeutic effects of hUCMSCs, we injected hUCMSCs (2 × 10^6^ cells) subconjunctivally or intravenously (IV) in the EAC mouse model at day 10 post-immunization (Fig. 1A). We observed typical allergic symptoms induced in the EAC mice after the SRW pollen challenge. Treatment by subconjunctival injection of hUCMSCs significantly alleviated allergic symptoms (Fig. 1B). However, IV administration of hUCMSCs and hUCMSC-CM did not attenuate the symptoms of EAC.

The severity of EAC was graded by the modified Magone scoring standard [25], including conjunctival redness (0–3), chemosis (0–3), lid edema (0–3), and tearing (0–3; total score: 0–12) (Fig. 1C). As compared with the EAC groups, the subconjunctival injection of hUCMSCs significantly reduced scratching behavior and clinical scores of allergy at all-time points, and hUCMSC-CM did not show a benefit in therapeutic outcome. The IV infusion of hUCMSCs had lower scores and less scratching behavior than the EAC groups at several time points, but the differences were not significant, and hUCMSC-CM administration did not yield therapeutic effects. The glucocorticoid (DEX) treatment resulted in the best therapeutic outcome (Fig. 1D). The above results demonstrate that subconjunctival injection of hUCMSCs significantly attenuated allergy scores and instances of scratching. We further confirmed that IV-infused hUCMSCs did not migrate to the conjunctiva (Additional file 3: Fig. S2). Therefore, we focused on the subconjunctival injection route in subsequent experiments.

### Subconjunctival injection of hUCMSCs reduced the inflammatory cells in EAC mice

**Fig. 2:**
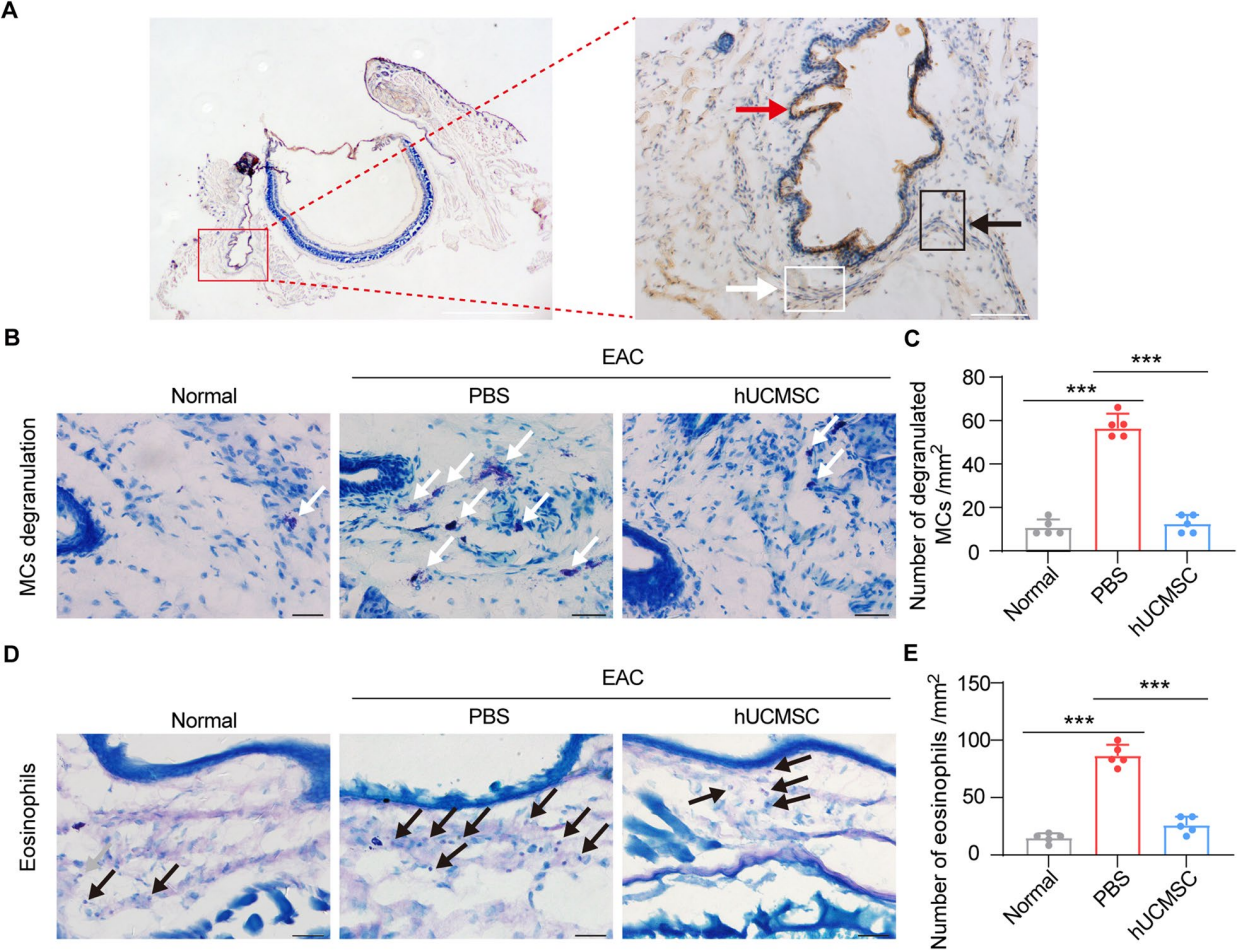
Subconjunctival administration of hUCMSCs reduces inflammatory cells in the conjunctiva. **A** Representative images of Cytokeratin 7 staining demonstrated the whole conjunctival epithelium (pointed by a red arrow). The area pointed by a white arrow was used for toluidine blue staining, and the area pointed by the black arrow was used for giemsa staining. **B** Representative images of MCs stained with toluidine blue in the conjunctiva. Degranulated MCs were noticeable in the EAC group but negligible in the other groups. The white arrows pointed to the degranulated MCs with a dark blue color surrounded by many small dark particles. Scale bar = 50 μm. **C** The quantitative analysis of the number of degranulated MCs per mm^2^ (*n* = 5). **D** The infiltrated eosinophils stained with giemsa in the subconjunctival space at day 15 post-SRW pollen challenge in different groups. The black arrows pointed to the eosinophils in pink. Scale bar = 50 μm. **E** The quantitative analysis of the number of eosinophils per mm^2^ (*n* = 5). Results were expressed as the mean ± SD, ***P < 0.001

Activated MCs and infiltrated eosinophils in the conjunctiva release inflammatory mediators that aggravate allergy symptoms in the late phases of AC [26]. We performed histopathological tests to evaluate whether hUCMSCs can reduce these cells in the conjunctiva (Fig. 2A). Toluidine blue staining revealed a higher number of activated degranulated MCs in the conjunctiva of EAC mice compared to the normal group (Fig. 2B, C), whereas the number of degranulated MCs was significantly decreased in the hUCMSC treatment group. In addition, giemsa staining showed that the number of eosinophils in the conjunctiva was reduced in hUCMSC-treated mice compared to the PBS group in the EAC mouse model (Fig. 2D, E). These findings indicate that hUCMSC treatment significantly reduced the presence of degranulated MCs and infiltrated eosinophils in the conjunctiva of EAC mice.

### hUCMSCs reduced Th2 cells in CLNs of EAC mice

**Fig. 3:**
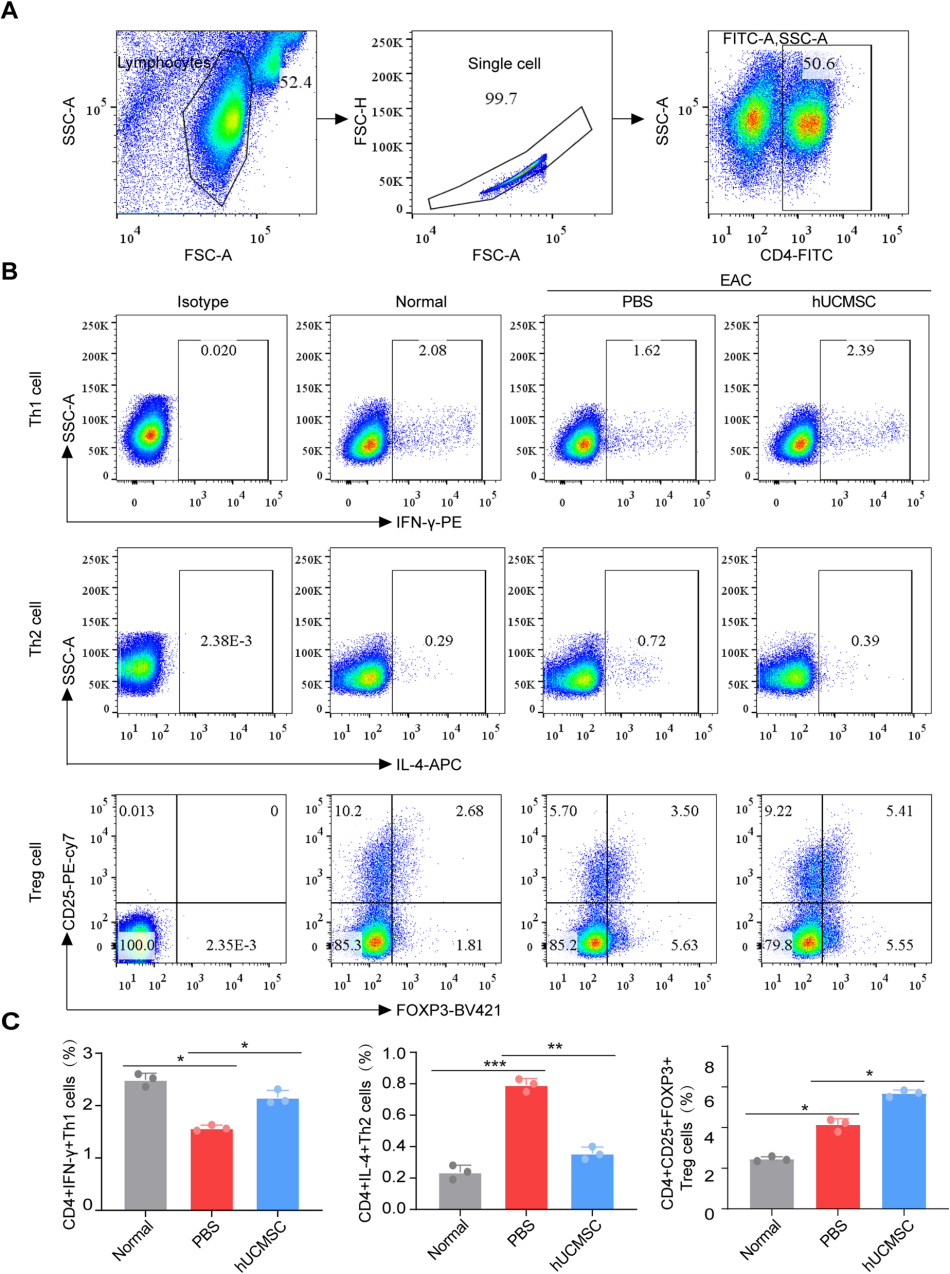
hUCMSCs change CD4 + T lymphocyte subsets in CLNs that are detected by flow cytometry. **A** CD4 + T cells in CLNs were gated by flow cytometry. **B** The proportion of Th1, Th2, and Treg cells among CD4 + T cells in CLNs was analyzed by flow cytometry. **C** Quantitative analysis of the proportion of Th1, Th2, and Treg cells among CD4 + T cells (*n* = 3). The data are expressed as mean ± SD, *P < 0.05, **P < 0.01

In the early phase of AC, the excessive activation of Th2 cells has been recognized to play a critical role. Th0 cells proliferate and differentiate into Th2 cells, with the latter causing eosinophilic infiltration by migrating to the conjunctiva and producing IL-4, IL-5, and IL-13. To further investigate the immunomodulatory effects of hUCMSCs on T cell phenotypes in the draining CLNs of EAC mice, we assessed the Th1 and Th2 cell populations and the number of Treg cells by flow cytometry. Results showed a lower ratio of CD4 + IFN-γ + Th1 cells, a high percentage of CD4 + IL-4 + Th2 cells, and CD4 + CD25 + FOXP3 + Treg cells in the CLNs in EAC mice compared with the control group. However, after treatment with hUCMSCs, an increased ratio of Th1 cells, a reduced ratio of Th2 cells, and an increased population of CD4 + CD25 + FOXP3 + Treg cells were observed in draining CLNs (Fig. 3A–C).

**Fig. 4:**
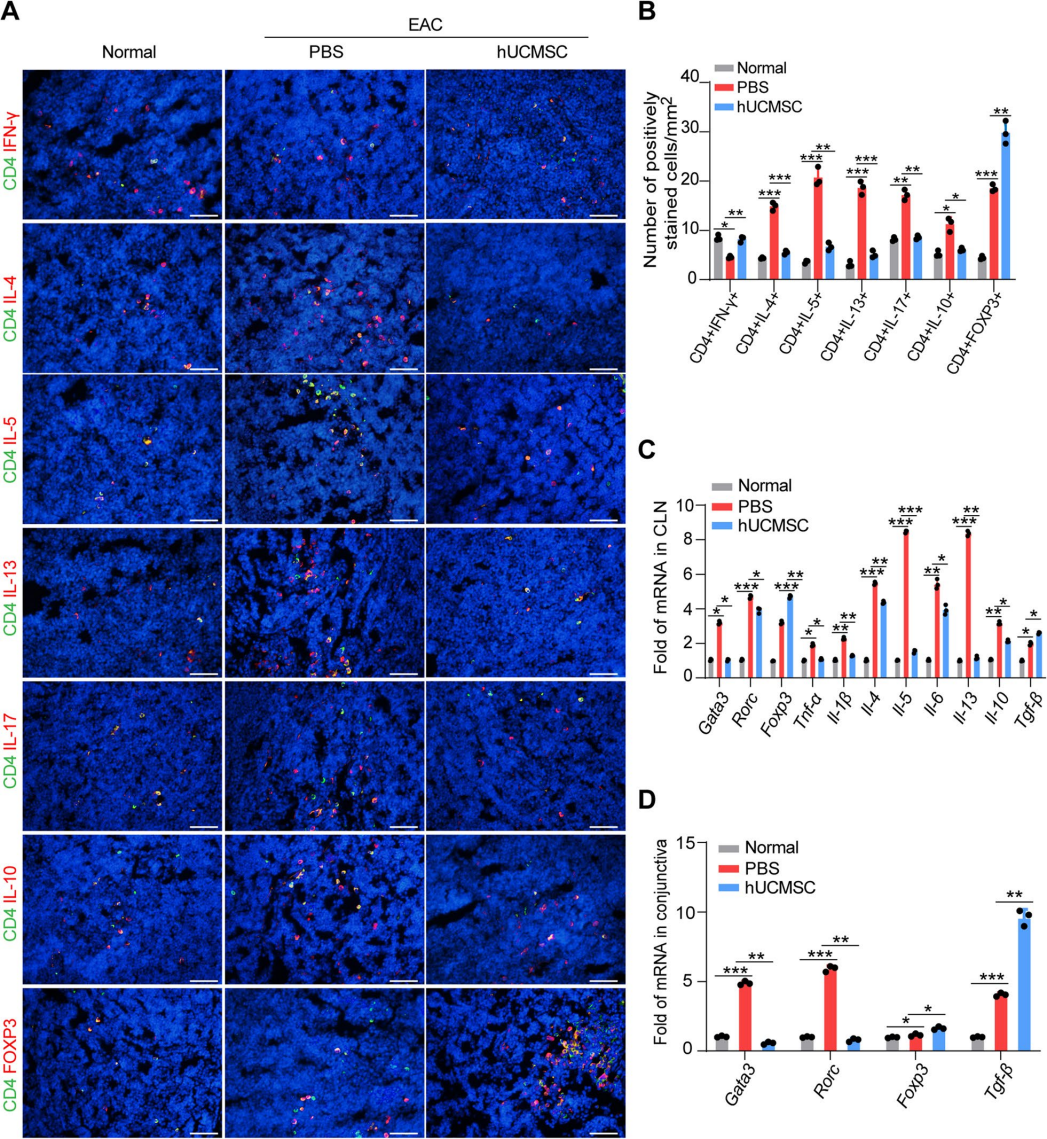
hUCMSCs change CD4 + T lymphocyte subsets that are detected by immunostaining and RT-qPCR. **A** The representative images of Th1 (IFN-γ), Th2 (IL-4, IL-5, IL-13), Th17 (IL-17), and Treg cells (FOXP3) in CLNs. Double staining showed increased Th2, Th1, Treg, and Th17 cells in CLNs of EAC. Scale bar = 50 μm. **B** Quantitative analysis of the number of Th1, Th2, Th17, and Treg cells per field (*n* = 3). **C** The expression levels of cytokines in CLNs (*n* = 3). **D** The markers of Th2, Th17, and Treg cells in the conjunctiva were quantitatively analyzed by RT-qPCR (*n* = 3). The data are expressed as mean ± SD, *P < 0.05, **P < 0.01, ***P < 0.001

In addition, immunofluorescent staining showed increased IL-4 + , IL-5 + , and IL-13 + T cells in the draining CLNs in EAC mice, demonstrating stronger Th2-dominant inflammation. In addition, Th17 cells derived from Th0 cells produce IL-17, which aggravates Th2-dominant allergic inflammation in allergic eye disease [27]. The number of Th17 cells was increased in CLNs in EAC mice (Fig. 4A, B). Subconjunctival injection of hUMCSCs markedly reduced the number of Th2 and Th17 cells. Furthermore, IFN-γ + Th1 cells were decreased in EAC mice and significantly increased with hUCMSC treatment. In addition, the levels of the transcription factor FOXP3 was increased in the draining CLNs of EAC mice and further increased in the hUCMSC treatment group. The expression levels of cytokines were confirmed by qRT-PCR (Fig. 4C). These findings collectively indicate that hUCMSCs markedly reduced the number of Th2 and Th17 immune cells and elevated the number of Treg cells in CLNs, thereby decreasing the levels of inflammatory factors, and increasing the levels of anti-inflammatory factors in draining CLNs.

We further examined the expression levels of Th2 marker *Gata3* and Th17 marker *Rorc* in conjunctiva tissues by real-time PCR and found that these two genes were upregulated in EAC mice compared to the control. Meanwhile, the expression of anti-inflammatory factors *Foxp3* and *Tgf-β* were increased in EAC mice. Administration of hUCMSCs significantly reduced the expression of *Gata3* and *Rorc*, and further increased the levels of anti-inflammatory factors *Foxp3* and *Tgf-β* (Fig. 4D). These results suggest that the local subconjunctival administration of hUCMSCs is able to reduce the number of Th2 and Th17 cells, and augment Treg cell population.

### hUCMSCs use CRISPLD2 to suppress the development of Th2 cells

**Fig. 5:**
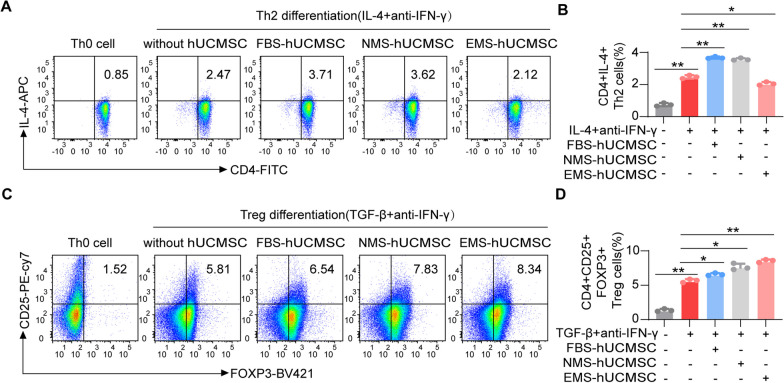
hUCMSCs inhibit the differentiation of Th0 into Th2 cells. hUCMSCs pretreated with EAC mouse serum (EMS-hUCMSCs) were co-cultured with Th0 in vitro, and the hUCMSCs pretreated with FBS (FBS-hUCMSCs) or normal murine serum (NMS-hUCMSCs) were used as controls. **A** EMS-hUCMSCs inhibited the differentiation of Th0 into Th2 cells positive for CD4 and IL-4. **B** Quantitative analysis of the IL-4 + Th2 cells in the indicated groups (*n* = 3). **C** EMS-hUCMSCs promoted the differentiation of Th0 into Treg cells positive for CD4, CD25 and FOXP3. **D** Quantitative analysis of the CD4 + CD25 + FOXP3 + Treg cells in the indicated groups (*n* = 3). The data are expressed as mean ± SD, *P < 0.05, **P < 0.01

It has been demonstrated that, in a pro-inflammatory environment, MSCs will exhibit an anti-inflammatory phenotype and secrete immunosuppressive factors [28]. Considering that immune cells will produce inflammatory factors and release them into the blood, we treated hUCMSCs with serum from EAC mice to mimic the in vivo EAC milieu and to determine whether hUCMSCs can regulate the differentiation and cytokine production of Th2 cells directly. We pretreated hUCMSCs with different kinds of serums and co-cultured them with freshly isolated Th0 cells. Five days later, Th2 (CD4 + IL-4 +) and Treg (CD4 + CD25 + Foxp3 +) cells generated in co-cultures were evaluated by flow cytometry. The results show that hUCMSCs treated with EAC mouse serum (EMS-hUCMSCs) repressed Th0 differentiation into Th2 lymphocytes (Fig. 5A, B). In addition, EMS-hUCMSCs also increased the generation of anti-inflammatory Treg lymphocytes versus hUCMSCs pretreated with FBS (FBS-hUCMSCs) or normal mouse serum (NMS-hUCMSCs) (Fig. 5C, D). These results indicate that hUCMSCs attenuate the severity of EAC through direct regulation of T cell differentiation.

**Fig. 6:**
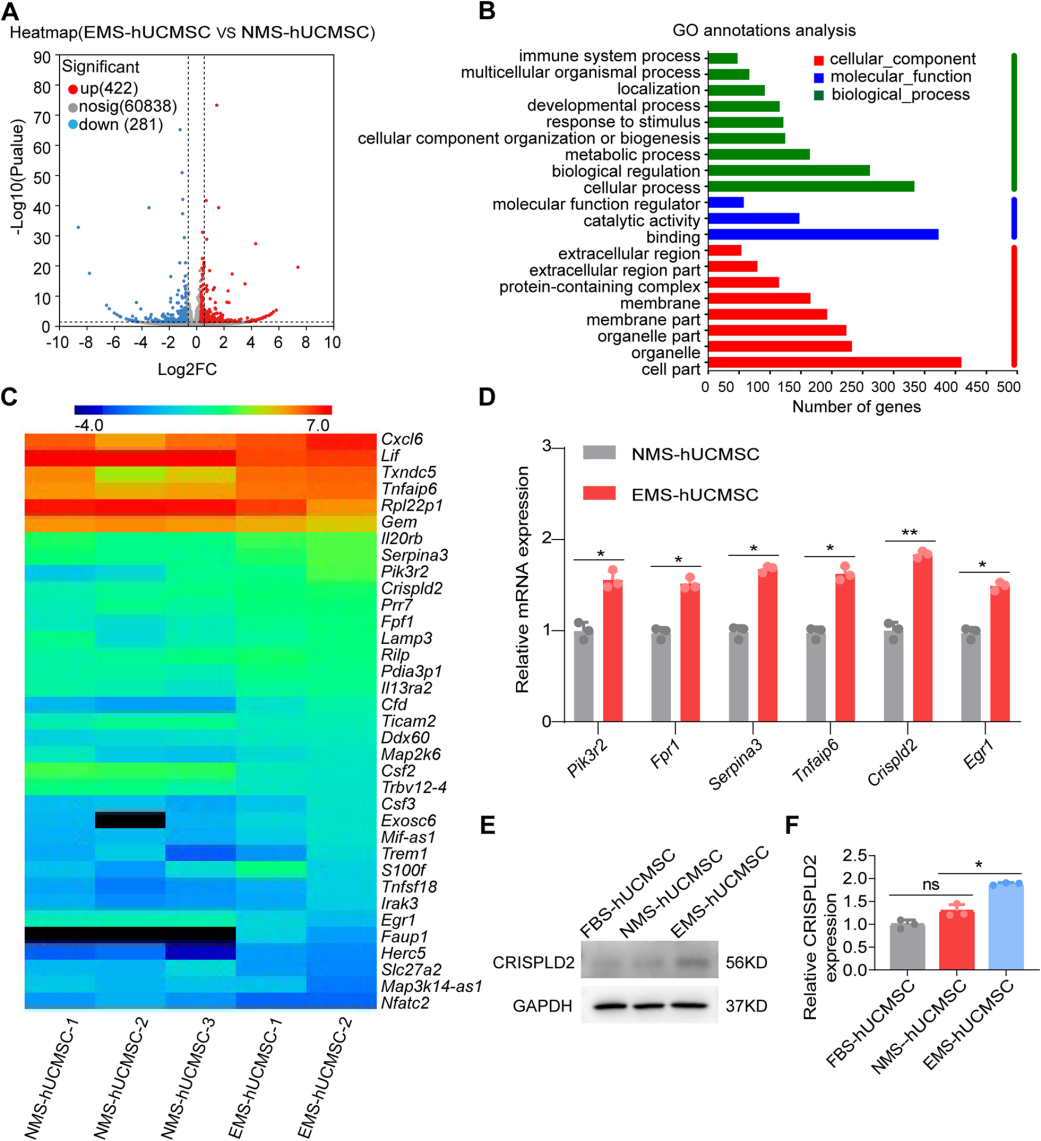
EMS-hUCMSCs express a higher level of the immunoregulatory factor *crispld2* than NMS-hUCMSCs. **A** A volcano plot of DEGs (EMS-hUCMSCs vs. NMS-hUCMSCs) that are upregulated (red) or downregulated (blue). **B** Go annotation analysis of DEGs (EMS-hUCMSCs vs. NMS-hUCMSCs) that are upregulated (red) or downregulated (blue). **C** A heatmap of the immune-related DEGs (EMS-hUCMSCs vs. NMS-hUCMSCs). **D** The immune-related DEGs were confirmed by qRT-PCR (*n* = 3). **E** Western blot was used to evaluate the expression of CRISPLD2 protein in EMS-hUCMSCs and NMS-hUCMSCs, full-length blots are presented in Additional file 4: Fig. S3. **F** Quantitative analysis of the expression level of CRISPLD2 (*n* = 3). The data are expressed as mean ± SD, *P < 0.05, **P < 0.01

Previous studies have identified several immunosuppressive factors in MSCs, including TSG6, PGE2, IDO, and TGF-β [1, 23]. To identify more factors that mediate the immunoregulatory capacity of hUCMSCs, we performed RNA sequencing of EMS-hUCMSCs and NMS-hUCMSCs. Of the 703 DEGs identified based on the threshold criteria, 281 genes were downregulated and 422 genes were upregulated in EMS-hUCMSCs versus NMS-hUCMSCs (Fig. 6A). GO annotations show that genes related to immune processes were enriched from the DEGs (Fig. 6B), represented by the heat map (Fig. 6C). Among them, six genes with high TPM values were verified by qRT-PCR (Fig. 6D). We decided to focus on *Crispld2*, as it demonstrated the greatest difference between the two groups. CRISPLD2 is a cystine-rich secretory protein (CRISP) that can inhibit LPS-mediated inflammation. Its features include a secretory signal and two LCCL domains [29]. The higher levels of CRISPLD2 in EMS-hUCMSCs compared to NMS-hUCMSCs were confirmed by Western blot (Fig. 6E, F).

**Fig. 7:**
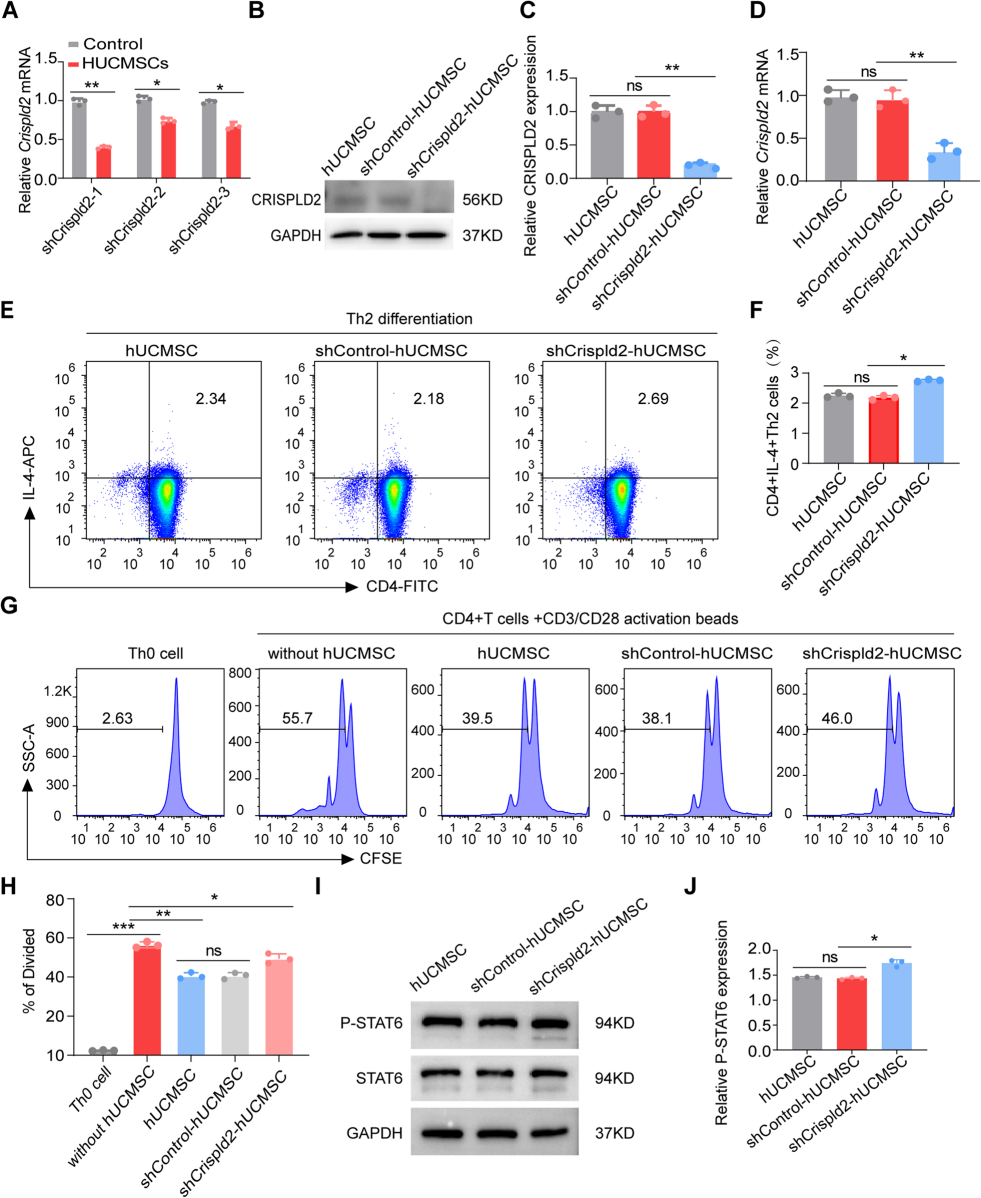
CRISPLD2 mediates hUCMSC repressing the differentiation of Th0 cells into Th2 cells. **A** qRT-PCR analysis of *crispld2* knockdown efficiency in hUCMSCs transfected with shRNA for the non-targeting sequence (shControl-hUCMSCs) or shRNA for the *crispld2* sequence (shCrispld2-hUCMSCs). **B** The protein level of CRISPLD2 was detected by Western blot, full-length blots are presented in Additional file 4: Fig. S3. **C** Quantitative analysis of the expression level of CRISPLD2 (*n* = 3). **D** qRT-PCR was performed to evaluate *crispld2* mRNA expression in shCrispld2-hUCMSCs treated with EAC mouse serum (*n* = 3). **E**, **F** The proportion of CD4 + IL-4 + cells were analyzed by flow cytometry **E** and quantitative analysis **F** in different groups (*n* = 3). **G**, **H** CD4 + T cells were labeled with CFSE, and the proliferation of CD4 + T cells was analyzed by flow cytometry **G** and quantitative analysis **H** in different groups (*n* = 3). **I** The protein expression of phosphorylated and total STAT6 in CD4 + T cells was detected by Western blot, full-length blots are presented in Additional file 4: Fig. S3. **J** Quantitative analysis of the expression level of p-STAT6 (*n* = 3). The data are expressed as mean ± SD, *P < 0.05, **P < 0.01, and ***P < 0.001

To investigate the biochemical mechanisms behind the regulation of Th2 differentiation by CRISPLD2, hUCMSCs were transfected with lentiviral particles to establish *Crispld2*-knockdown cells. We constructed three specific shRNA vectors targeting *Crispld2* and verified their knockdown efficiency by qRT-PCR (Fig. 7A). Based on the results, we selected shCrispld2-1 and transfected hUCMSCs (shCrispld2-hUCMSCs) for further investigations. The effective knockdown of shCrispld2-hUCMSCs was further confirmed by Western blot and qRT-PCR (Fig. 7B–D). To investigate the role of CRISPLD2 in hUCMSC-mediated immunomodulation, we co-cultured Th0 cells for 5 days in the presence of Th2 differentiation conditions, together with shCrispld2-hUCMSCs pretreated with EAC mouse serum. FACS results show that the IL-4 + CD4 + Th2 cell population was markedly increased when co-cultured with shCrispld2-hUCMSCs compared to the group co-cultured with shControl-hUCMSCs (Fig. 7E, F). These findings demonstrate that CRISPLD2 mediates the suppression of Th2 differentiation by hUCMSCs.

In order to explore the role of CRISPLD2 in hUCMSC-mediated immunosuppression, an in vitro T cell proliferation experiment was performed. Sorted CD4 + T cells were cultured in the presence of CD3/CD28 activation beads and labeled with CFSE. After being co-cultured with hUCMSCs, shControl-hUCMSC, or shCrispld2-hUCMSCs pretreated with EAC mouse serum for 3 days, the cells were collected and analyzed by flow cytometry. We found that hUCMSC and shControl-hUCMSC strongly suppressed the proliferation of activated CD4 + T cells (Fig. 7G, H), while shCrispld2-hUCMSCs showed less suppressive capacity. This further confirms that the immunosuppression function of hUCMSCs is partly mediated by CRISPLD2.

IL-4 is thought to promote Th2 cell differentiation by activating the STAT6 signaling pathway. To assess whether CRISPLD2 represses Th2 cell differentiation by inhibiting the STAT6 pathway, we performed Western blot analysis of phosphorylated STAT6 in activated T cells pretreated with EAC mouse serum and co-cultured with either shCrispld2-hUCMSCs or shControl-hUCMSCs (Fig. 7I, J). Compared with the shControl-hUCMSCs, shCrispld2-hUCMSCs markedly increased the expression of phosphorylated STAT6 induced by IL-4. This finding suggests that CRISPLD2 mediates the differentiation of Th2 cells by inhibiting STAT6 phosphorylation.

## Discussion

In this study, we found that subconjunctival injection of hUCMSCs strongly suppressed conjunctival inflammation and alleviated allergy symptoms compared to intravenous injection of hUCMSCs in a mouse EAC model. The therapeutic effects of hUCMSCs involve significant inhibition of Th2 cell differentiation in conjunctival tissue and draining CLNs, mediated by CRISPLD2.

Although the most common route for MSC application is intravenous injection in the treatment of systemic diseases, local administration avoids the ‘first-pass’ accumulation of MSCs in the lungs. Conjunctival injection delivers a high effective cell concentration into the space between the conjunctiva and Tenon’s capsule with fewer systemic side effects, and immediately produces paracrine signaling factors to maintain tissue homeostasis [30]. Studies have reported that among topical, subconjunctival, intravenous, and intraperitoneal routes, subconjunctival administration is the most effective for ocular repair [31]. In our mouse EAC model, most of the intravenously infused hUCMSCs may have accumulated in the lungs and left nearly no cells to migrate to the conjunctiva, thereby yielding no therapeutic effect. Topical and intraperitoneal routes may also fail to accumulate in the conjunctiva. The injected hUCMSCs are unlikely to migrate from the conjunctiva to the submandibular lymph nodes. However, the lymphocytes in the eyelids are drained into the preauricular node, parotid, or submandibular nodes [32]. The lymphocytes in the conjunctiva might be regulated by hUCMSCs and return to the cervical lymph nodes. In addition, it is possible that hUCMSCs in the conjunctiva release immunoregulatory factors that are transferred to the cervical lymph nodes via the lymphatic circulation, thus playing a regulatory role in the differentiation of T cells therein. Furthermore, the presence of a large number of T cells in the lymph nodes makes it easy to obtain enough T cells to determine the regulatory role of hUCMSCs. Therefore, subconjunctival administration of hUCMSCs demonstrated better therapeutic outcomes for AC treatment.

Th0 cells, under different disease conditions, differentiate into Th1, Th2, Th17, and Treg cells. Meanwhile, MSCs are involved in regulating T cell differentiation by producing a wide array of cytokines and growth factors. For instance, MSCs inhibit Th17 differentiation by IDO and PD-1, promote Treg differentiation by TGF-β and IL-10, and inhibit T cell proliferation by HLA-G1, TGF-β, and HGF [33–35]. Th2 cells play an important role in allergic diseases. Peng et al. demonstrated that MSC-derived small extracellular vesicles suppressed Th2 responses in patients with allergic rhinitis [36]. However, IDO was reported to induce the differentiation and maturation of Th2 cells. Furthermore, we found that hUCMSCs effectively promote Th2 cell differentiation when co-cultured with Th0 cells. Nonetheless, pretreatment with the serum of EAC mice enabled hUCMSCs to inhibit Th2 cell differentiation. This finding coincides with a previous report that serum from asthmatic mice potentiates the therapeutic effects of MSCs in experimental allergic asthma and increased the expression of various mediators, including TGF-β, IFN-γ, IL-10, TSG-6, IDO-1, and IL-1RN [37]. These results confirm that certain conditions are required for MSCs to inhibit allergic inflammation. For instance, the CM from bone marrow-derived MSCs stimulated by TNF-α was able to alleviate EAC [18]. Indeed, hUCMSCs not only reduced the number of Th2 cells, but also increased the number of Th1 cells in vivo, which suggests that a suitable ratio of Th1/Th2 is important for maintaining tissue homeostasis.

Notably, serum from EAC mice led to higher expression of CRISPLD2 in hUCMSCs compared to those unstimulated or stimulated with serum from normal mice. CRISPLD2, a cystine-rich secretory protein with a secretory signal and two LCCL domains, competitively inhibits LPS binding to its original target receptor TLR4, thereby reducing LPS-derived inflammation. Endogenous CRISPLD2 in healthy human serum was found to downregulate LPS-induced TNF-α production in vitro, and CRISPLD2 was found to protect mice from endotoxic shock [38]. In addition, CRISPLD2 also exhibits a broad range of anti-inflammatory properties in a series of inflammation-related diseases, including asthma and obesity. Dexamethasone and IL1-β can also elevate CRISPLD2 levels, which then modulate the expression of inflammatory genes IL-6 and IL-8 [28]. Another study reported that CRISPLD2 attenuates pro-inflammatory cytokines TNF-α, IL-6, IL-8, and MCP-1 production in HMGB1-stimulated monocytes and septic mice [39]. The co-culturing of hUCMSCs with Th0 cells demonstrates that CRISPLD2 was able to inhibit Th2 differentiation.

Several reports have shown that, following activation of Janus-kinases (JAKs) in allergic conjunctivitis, IL-4 and IL-13 induce the expression of specific genes by activating STATs [40, 41]. STAT6 signaling has been recognized as a necessary part of developing the Th2 response and allergic inflammation [42]. The inhibition of STAT6 activities by CRISPLD2 suggests a potential mechanism through which the suppression of the JAK/STAT signaling pathway results in the repression of Th2 cell differentiation. Previous work reported that CRISPLD2 inhibited the TLR4 signaling pathway [43]. However, since activation of TLR4 inhibits the expression of STAT6 [44], it is obvious that CRISPLD2 repressed STAT6 phosphorylation is not mediated by the TLR4 signaling pathway. Therefore, more efforts are needed to reveal the target and mechanism of CRISPLD2 in regulating T cell differentiation.

This study is limited by the wide scope of synergistic actions between reported factors and novel mediators from hUCMSCs that mediate therapeutic functions, rather than one or two modulators. Among the other upregulated molecules (Fig. 6D), Phosphoinositide-3-Kinase Regulatory Subunit 2 (PIK3R2) has the capacity to repress T cell proliferation in mice [45, 46]; formyl peptide receptor 1 (FPR1) is expressed in various immune cells and has a complex role in pathogenic processes [47]. Tumor necrosis factor-induced protein 6 (TNFAIP6) secreted by bone marrow–derived MSCs attenuates inflammatory bowel disease by modulating follicular helper T cells and follicular regulatory T cells balance in mice [48]. Hence, further studies are needed to identify and characterize factors in the hUCMSC secretome. These factors have the potential to be utilized as topical immunosuppressive formulations for AC.

## Conclusion

Our results demonstrate that subconjunctival injection of hUCMSCs significantly suppressed conjunctival inflammation and alleviated allergy symptoms in an SRW-induced mouse EAC model. After pretreatment with serum from EAC mice, hUCMSCs repressed the differentiation of Th2 cells, modulating the Th1/Th2 balance. In addition, EAC mouse serum-exposed hUCMSCs were found to be potent promoters of Treg cell differentiation and activation. We further confirmed that these immunomodulatory effects of hUCMSCs were mediated, at least partly, by CRISPLD2. This study not only provides a potential therapeutic target for the treatment of allergic conjunctivitis but also other T cell-mediated diseases.

### Supplementary Information

**Additional file 1**. **Table S1:** Antibodies for identifying the differentiation of Th0 into Th2 cells. **Table S2:** Primers for analysis of expression levels of the cytokines and the differentiation-related genes of Th0 cells.

**Additional file 2**. **Figure S1**: hUCMSCs identification. (A) Morphology (Bright field), adipogenesis (Oil-red-O), osteogenesis (AKP), and chondrogenesis (Toluidine blue) of hUCMSCs. (B) Flow cytometry analysis of cell membrane markers on hUCMSCs. Scale bar = 50 μm.

**Additional file 3**. **Figure S2**: hUCMSCs are not able to migrate to the conjunctiva by intravenous infusion. The lentiviral pLVX-shRNA2-ZsGreen1 (Takara) vector was used to prepare lentivirus and infect hUCMSCs. The positively transfected cells were sorted by FACS based on ZsGreen expression (green color), and the ZsGreen-labeled hUCMSCs (2×106 cells) were intravenously or subconjunctivally injected into the mouse EAC model. 24 hours later, samples were collected and used for preparing cryosections. hUCMSCs did not migrate to the conjunctiva after intravenous infusion. Scare bar = 50μm.

**Additional file 4**. **Figure S3**: Original images of Western blot.

## Data Availability

All data generated or analyzed during this study are included in this published article and its supplementary information files. The raw data of RNA-seq have been deposited in the NCBI Sequence Read Archive (SRA) database under accession number: PRJNA931840.

## Abbreviations

AC: Allergic conjunctivitis
ARVO: Association for Research in Vision and Ophthalmology
CFSE: Carboxy-fluorescein succinimidyl ester
CLNs: Cervical lymph nodes
CRISPLD2: Cystine-rich secretory protein (CRISP) and two LCCL domains
DMEM: Dulbecco’s modified Eagle medium
EDTA: Ethylene diamine tetraacetic acid
EAC: Experimental allergic conjunctivitis
FOXP3: Forkhead box protein 3
hUCMSCs: Human umbilical cord mesenchymal stem cells
MCs: Mast cells
MACS: Magnetic cell separation
SRW: Short ragweed pollen
STAT6: Signal transducer and activator of transcription 6
TNF-α: Tumor necrosis factor alpha
