# Supplementary figures and images for: Subconjunctival injection of human umbilical cord mesenchymal stem cells alleviates experimental allergic conjunctivitis via regulating T cell response
Source: Stem Cell Res Ther. 2023 Oct 2;14:281. doi: 10.1186/s13287-023-03484-4 (PMC10546642; doi:10.1186/s13287-023-03484-4)

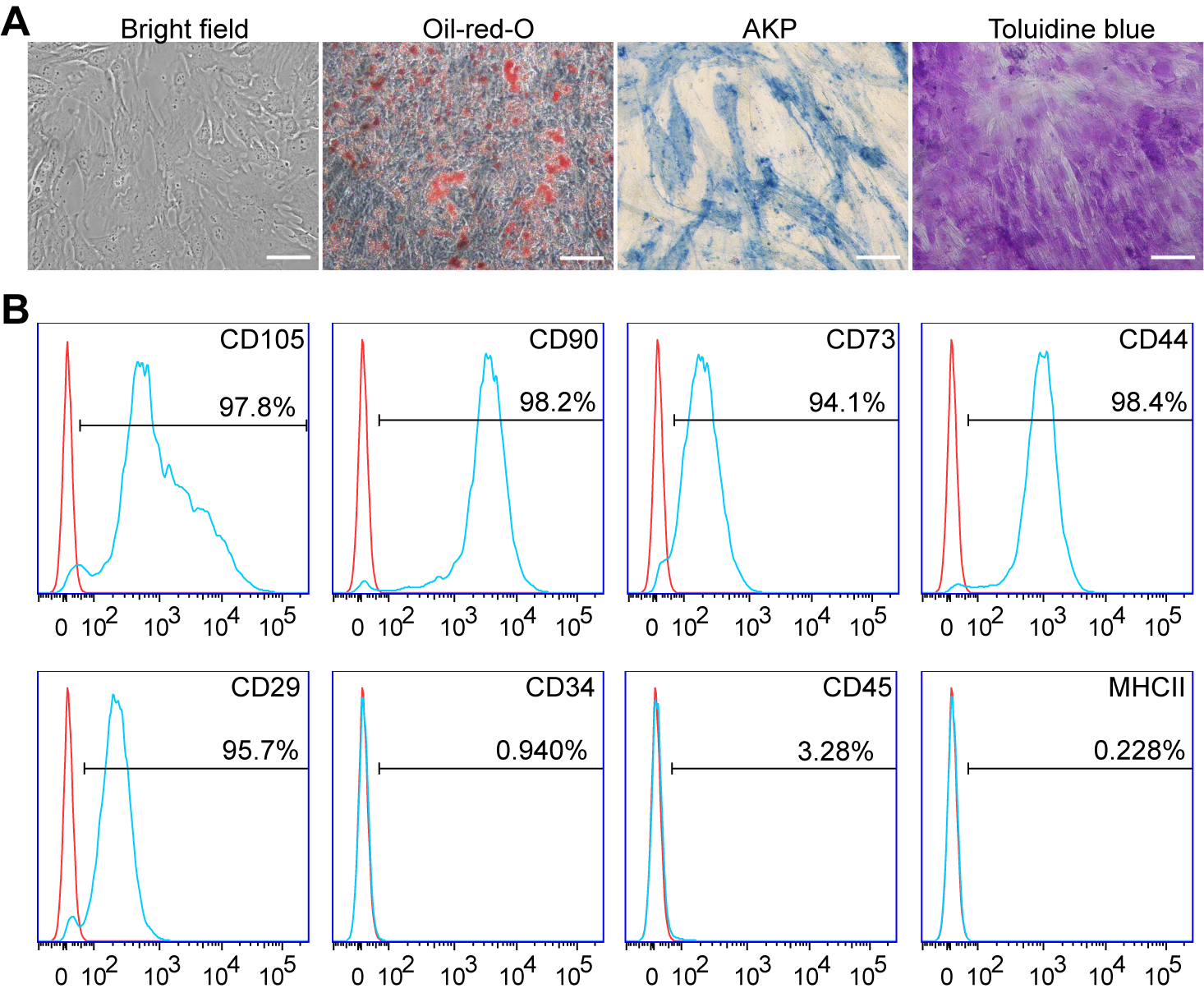

Supplement: Supplementary file 2 — Additional file 2. Figure S1: hUCMSCs identification. (A) Morphology (Bright field), adipogenesis (Oil-red-O), osteogenesis (AKP), and chondrogenesis (Toluidine blue) of hUCMSCs. (B) Flow cytometry analysis of cell membrane markers on hUCMSCs. Scale bar = 50 μm. [file 13287_2023_3484_MOESM2_ESM.tif]

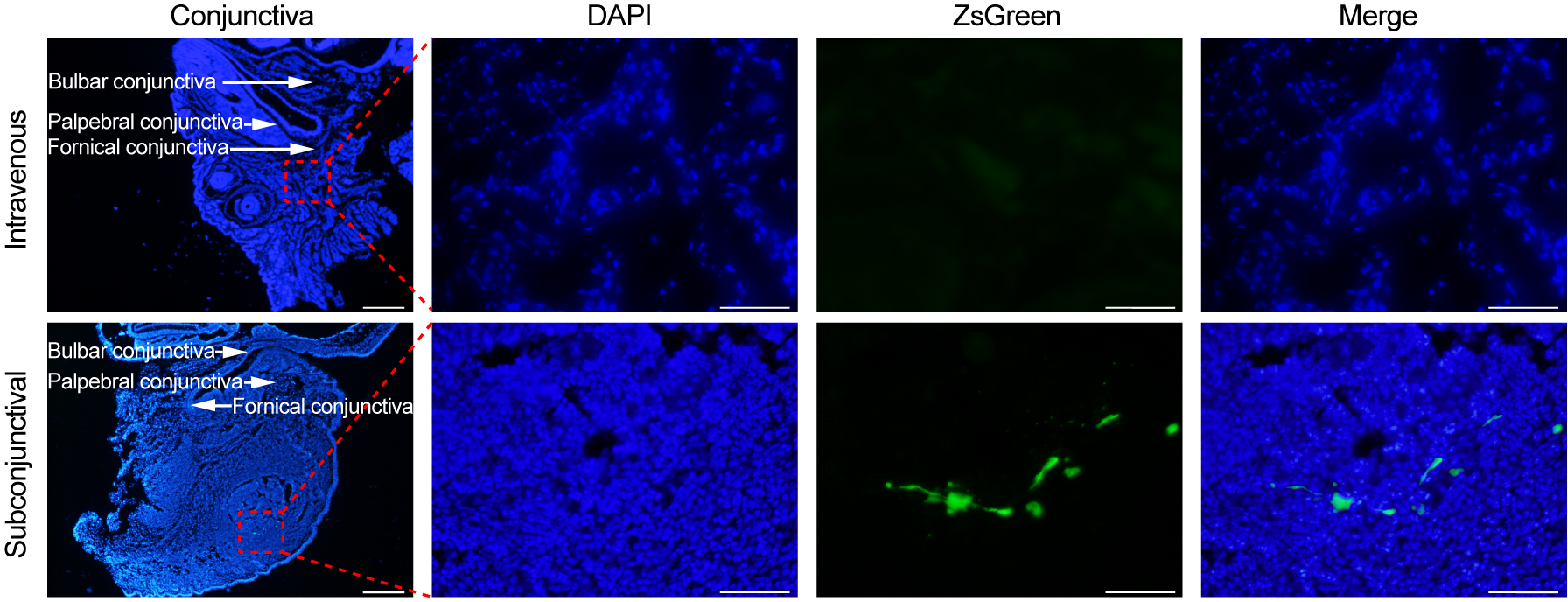

Supplement: Supplementary file 3 — Additional file 3. Figure S2: hUCMSCs are not able to migrate to the conjunctiva by intravenous infusion. The lentiviral pLVX-shRNA2-ZsGreen1 (Takara) vector was used to prepare lentivirus and infect hUCMSCs. The positively transfected cells were sorted by FACS based on ZsGreen expression (green color), and the ZsGreen-labeled hUCMSCs (2×10^6^ cells) were intravenously or subconjunctivally injected into the mouse EAC model. 24 hours later, samples were collected and used for preparing cryosections. hUCMSCs did not migrate to the conjunctiva after intravenous infusion. Scare bar = 50μm. [file 13287_2023_3484_MOESM3_ESM.tif]

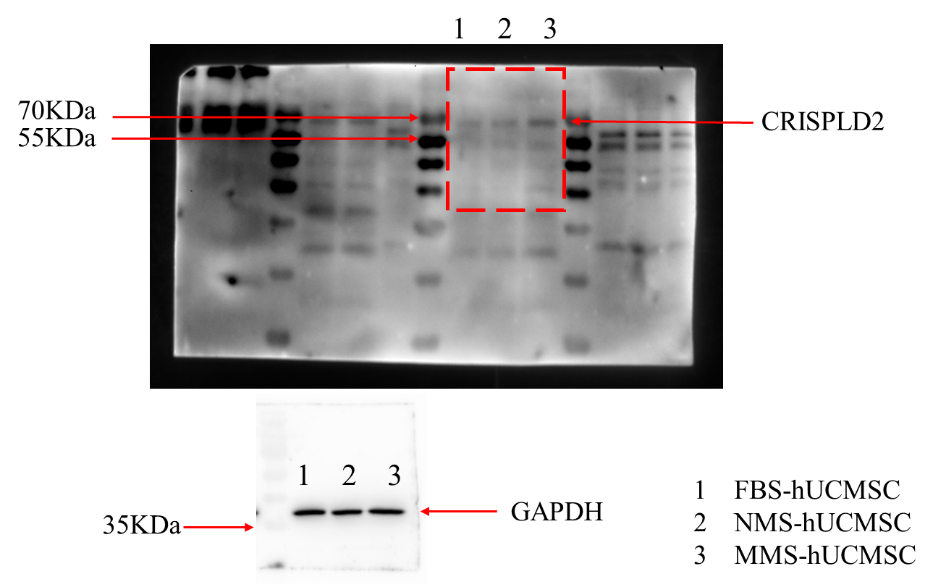
 Figure 6E

Figure 7B


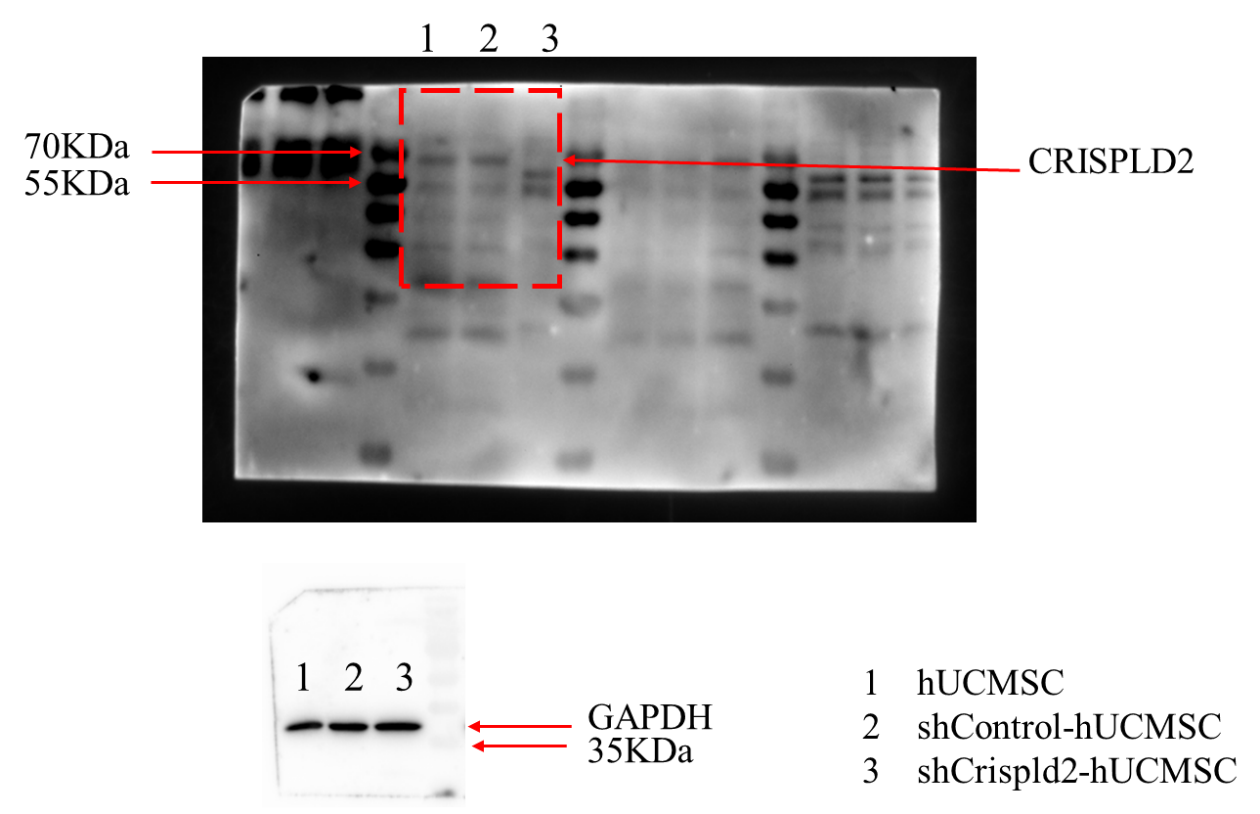


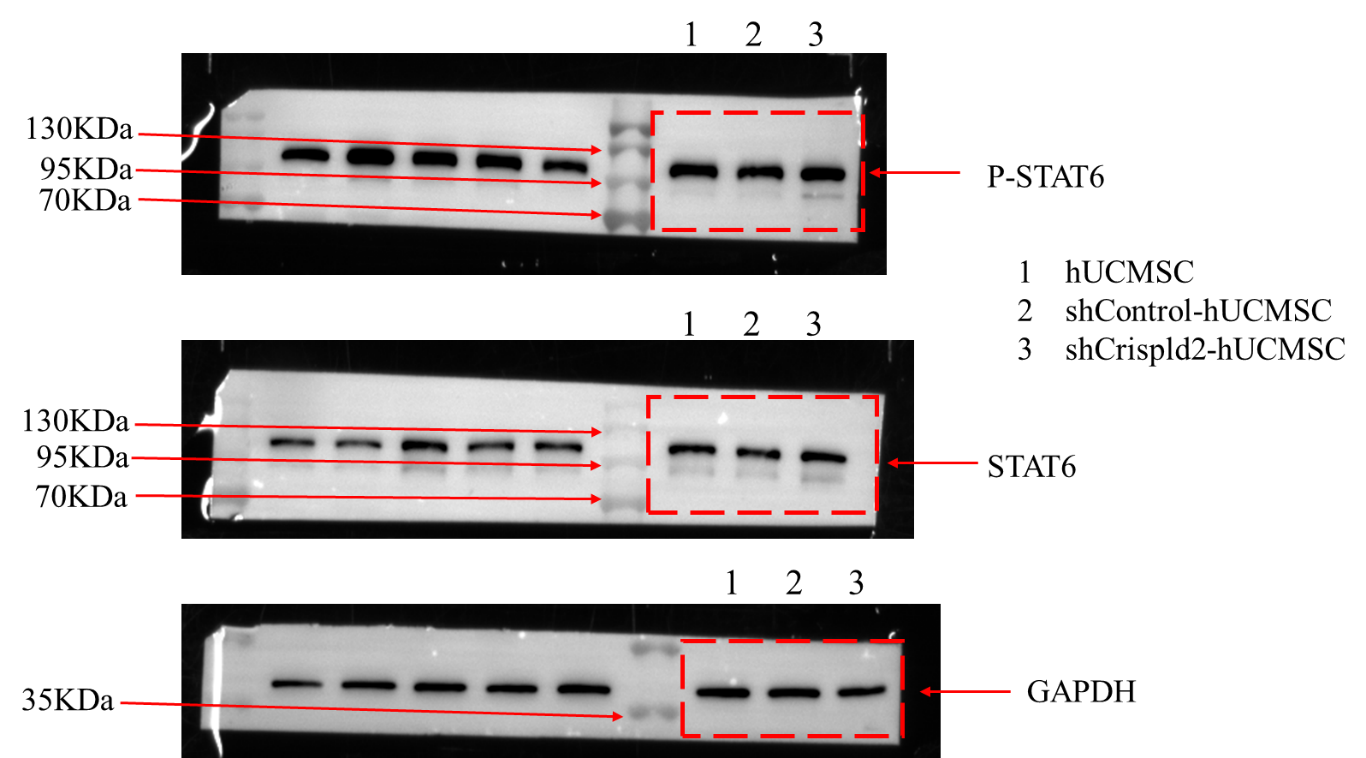
 Figure 7I

Supplement: Supplementary file 4 — Additional file 4. Figure S3: Original images of Western blot. [file 13287_2023_3484_MOESM4_ESM.docx]
